# In Patients With Multiple Sclerosis, Both Objective and Subjective Sleep, Depression, Fatigue, and Paresthesia Improved After 3 Weeks of Regular Exercise

**DOI:** 10.3389/fpsyt.2019.00265

**Published:** 2019-05-03

**Authors:** Dena Sadeghi Bahmani, Juerg Kesselring, Malamati Papadimitriou, Jens Bansi, Uwe Pühse, Markus Gerber, Vahid Shaygannejad, Edith Holsboer-Trachsler, Serge Brand

**Affiliations:** ^1^Center for Affective, Stress and Sleep Disorders, Psychiatric Clinics, University of Basel, Basel, Switzerland; ^2^Substance Abuse Prevention Research Center and Sleep Disorders Research Center, Department of Psychiatry, Kermanshah University of Medical Sciences, Kermanshah, Iran; ^3^Isfahan Neurosciences Research Center, Alzahra Research Institute, Isfahan University of Medical Sciences, Isfahan, Iran; ^4^Kliniken Valens, Valens, Switzerland; ^5^Sport Science Section, Department of Sport, Exercise and Health, University of Basel, Basel, Switzerland; ^6^Department of Neurology, School of Medicine, Isfahan University of Medical Sciences, Isfahan, Iran

**Keywords:** cognitive performance, exercise, rehabilitation, multiple sclerosis, physical activity intervention, sleep-EEG, sleep

## Abstract

**Background:** Multiple sclerosis (MS) patients suffer from various difficulties including sleep complaints, symptoms of depression and fatigue, paresthesia, and cognitive impairments. There is growing evidence that regular physical activity has a positive effect on both sleep and psychological functioning, though there is limited evidence of this kind for MS patients. The aim of the present study was therefore to investigate the impact on this patient group of a regular exercise program with respect to subjective and objective sleep, depression, paresthesia, fatigue, and cognitive performance.

**Methods:** A total of 46 patients [mean age: 50.74 years; Expanded Disability Status Scale (EDSS): mean: 5.3, 78.4% females] completed this 3-week intervention study. At baseline and 3 weeks later, they answered questionnaires covering sociodemographic information, subjective sleep, depression, fatigue, paresthesia, and subjective physical activity. Objective sleep [sleep electroencephalogram (EEG) recordings] and cognitive performance were also assessed at both time points. Patients participated in a regular exercise activity every weekday for about 60 min.

**Results:** Compared to the baseline, by the end of the study, objective sleep had significantly improved (sleep efficiency, sleep onset latency, and wake time after sleep onset), and symptoms of sleep complaints, depression, fatigue, and paresthesia were significantly reduced. Subjective physical activity (moderate and vigorous) and cognitive performance also increased over the course of the intervention.

**Conclusions:** In patients with MS, participation in regular exercise impacted positively on their objective and subjective sleep, depression, paresthesia, fatigue, and cognitive performance.

## Introduction

Worldwide, about 2.5 million people suffer from multiple sclerosis (MS) (1). Multiple sclerosis (MS) is defined as a neurodegenerative disease involving both psychological and physical impairments (2). Typically, MS sufferers have sleep complaints, symptoms of depression, fatigue, and paresthesia (3–8), reduced physical activity levels (9–13), and cognitive impairments (14–17). Importantly, Carta et al. (18) and Fornaro et al. (19) underlined the importance of a thorough pharmacological treatment of psychiatric symptoms in individuals with MS. With regard to sleep, recent research has shown that at MS onset, sleep is not impaired (20). The same research group also found that sleep quality in these patients remained unchanged 2 years after disease onset (21). Not surprising, poor sleep, symptoms of depression, paresthesia, fatigue, and cognitive impairments have a negative impact on quality of life (22, 23). It follows therefore that their improvements should have a beneficial effect on patients’ quality of life (24–26). There are various treatment options for improving attributes such as sleep and cognitive performance and reducing depression and fatigue, among MS patients, and given that these psychological and physical characteristics appear to be highly interlinked, one might expect that their improvements or impairments would also be associated with improvements or impairments in the others (27–31).

With regard to sleep, numerous studies of otherwise healthy individuals have shown poor sleep to be associated with poor emotion regulation (32–34), lower cognitive performance (35, 36), and mood alterations (37, 38). Among MS patients, poor sleep has been linked to impaired cognitive performance (28, 31), higher fatigue scores, and depression (27, 39, 40). Several reviews also conclude that sleep is impaired in about 50% of MS patients (3, 4, 6–8, 29, 41–43). However, Sadeghi Bahmani et al. (20) showed that, among a small sample of MS patients aged 32.3 years, sleep quality indices at disease onset did not differ from the indices of nonclinical adolescents and young adults. It is notable that in the same group of patients, sleep quality indices were no different 2 years later (21), though sedentary behavior had decreased and levels of moderate physical activity increased, but vigorous physical activity had decreased.

There are several potential treatment options for improving the sleep of MS patients. Pharmacological interventions mainly consist of the prescription of benzodiazepines and selective serotonin reuptake inhibitors (SSRIs), although their long-term efficacy appears to be questionable. Furthermore, their side effects can be irritating and may even increase daytime sleepiness, depression, and fatigue, and worsen cognitive performance (3, 44, 45); for these reasons, they are not suitable for long-term treatment. Nonpharmacological treatment approaches include cognitive behavioral therapy (46, 47) and relaxation techniques (43, 48–50). Several studies of healthy individuals have also shown that exercise interventions can improve sleep (51–55). However, there are only a few studies on the benefits of interventions for MS patients. As one example, Siengsukon et al. (56) randomly allocated 28 MS patients either to moderate-intensive aerobic exercise or to low-intensive walking/stretching. Patients engaged in one or other of these activities for 30 min three times a week, and for consecutive 12 weeks. Outcome variables were sleep quality, daytime sleepiness, fatigue, and depression. Results showed that sleep quality improved descriptively from baseline to postintervention. Nevertheless, a significant improvement in sleep was only observed in the low-intensity walking/stretching condition, whereas daytime sleepiness improved only in the moderate-intensity aerobic exercise condition. Interestingly, improvements in sleep and daytime sleepiness were not associated with any changes in depression or fatigue. To conclude, given the paucity of research into physical activity interventions to improve sleep among patients with MS, the first aim of the present study was to examine whether an intervention involving regular exercise could improve both objective and subjective sleep.

Additionally, there is a growing body of research showing that regular physical activity has a positive impact on fatigue, depression, and, for example, cardiovascular fitness in MS patients (10–12, 24, 57–63). In this regard, De Berardis et al. (64), Marini et al. (65), and Lang and Borgwardt (66) showed associations between symptoms of major depressive disorder and inflammatory markers.

With regard to paresthesia, to our knowledge, Razazian et al. (67) were first to show that regular physical activity (yoga and aquatic exercise) could reduce paresthesia, although indirectly *via* reductions in depression. Therefore, the second aim of the present study was to determine whether regular exercise would also have a positive impact on depression, fatigue, and paresthesia. Accordingly, the endpoints were as follows: Changes in objective and subjective sleep, depression, fatigue, and paresthesia. Secondary endpoint variables were physical activity and cognitive function.

Four hypotheses were formulated. First, following others (51, 52, 54), we expected that the objective sleep of MS patients would improve over 3 weeks of regular exercise. Second, following Siengsukon et al. (56), we anticipated that 3 weeks of regular exercise would improve their subjective sleep. Third, following others (10, 24, 57, 58, 67, 68), we hypothesized that 3 weeks of regular exercise would have a positive impact on depression, fatigue, and paresthesia among such patients. Fourth, in line with previous research (69–71), we expected that a 3-week program of regular exercise would improve the cognitive performance of MS patients.

## Methods

### Procedure

In the present study, inpatients with MS of the Klinik Valens (a rehabilitation center located in the Southeastern part of Switzerland) were approached and asked to participate in the present 3-week intervention study (a 3-week stay was the fixed duration of hospitalization in the clinic based on the insurance system in Switzerland). All participants were informed about the voluntary character of their participation and were assured that all the data were gathered anonymously. Signed written informed consent was obtained from the participants. All assessments took place both at baseline and 3 weeks later at discharge. At both time points, participants completed a series of questionnaires covering sociodemographic data, sleep complaints, depression, fatigue, paresthesia, and physical activity (see Tools below). Completion of the questionnaire took 30–40 min. A trained expert assessed participants’ cognitive performance and applied the EEG device for sleep assessment during one night at baseline and study end.

The local ethics committees of Basel (Ethikkommission Nordwestschweiz; EKNZ: 2016-01347) and St. Gallen (EKOS; Switzerland) approved the study, which was carried out in accordance with the ethical principles laid down in the Declaration of Helsinki and its later amendments.

### Participants

Fifty-one patients were consecutively selected. Details of their EDSS scores and disease duration data were extracted from the Klinik Valens registered data. Inclusion criteria were as follows: a) physician-determined diagnosis of MS (following the McDonalds’ diagnostic criteria for MS) (72) irrespective of subtype (relapse–remitting, primary progressive, and secondary progressive), b) neurologist-rated EDSS score below 6.0, c) age between 18 and 65 years, d) willing and able to comply with the study requirements as attested by signed written informed consent, e) able to read and write in German, and f) stable MS-related pharmacological treatment such as glatiramer acetate, interferons, fumarates and immune suppressiva, and monoclonal antibodies at the beginning of the study and throughout the whole study. Exclusion criteria were as follows: a) other neurological or severe psychiatric disorders and b) intake of medications apart from MS-related medications and antidepressants.

## Measures

### Objective Sleep

As in previous studies (73–77), we assessed objective sleep first for one night at baseline and then for one night at the end of the study using a one-channel, portable sleep-EEG recording device. Its validity has been repeatedly confirmed (77). The following sleep continuity parameters were measured: sleep onset latency, sleep period time, total sleep time, and number and time of awakenings after sleep onset. The following sleep architecture parameters were measured: awakenings after sleep onset (number and duration in minutes); (non-REM sleep) stages 1 and 2 (light sleep; min and %) and 3 and 4 (slow wave sleep/deep sleep; min and %); rapid eye movement sleep (REM; min and %); and REM latency (time between sleep onset and first appearance of REM sleep; min).

### Subjective Sleep

In line with previous research (78, 79), we measured sleep disturbances using the Insomnia Severity Index (ISI) (78). This questionnaire is a seven-item screening measure for insomnia and an outcome measure for use in treatment research. The items, answered on a five-point Likert scale ranging from 0 (not at all) to 4 (very much), refer in part to the *Diagnostic and Statistical Manual of Mental Disorders* (80) criteria for insomnia by assessing difficulty falling asleep, difficulty remaining asleep, early morning awakenings, impaired daytime performance, low satisfaction with sleep, and worry about sleep. The higher the overall score, the more the participant is assumed to suffer from insomnia. Cronbach’s α for the present sample was 0.87.

### Depressive Symptoms

To assess symptoms of depression, we used the Beck Depression Inventory–Fast Screen (BDI-FS) (81). The BDI-FS is a brief self-report inventory designed to evaluate depression in patients with medical illness. It consists of seven items, and every item has a set of four possible responses, representing different levels of symptom severity (e.g., or sadness: 0 = “I don’t feel sad”; 1 = “I feel sad”; 2 = “I’m sad all the time and I can’t snap out of it”; 3 = “I’m so sad/unhappy, that I can’t stand it”). Higher scores reflect a greater severity of depressive symptoms (range: 0–21). Cronbach’s α for the present sample was 0.90.

### Fatigue

Participants completed the Fatigue Severity Scale (FSS) (82). The FSS consists of nine items, and answers are given on seven-point rating scales ranging from 1 (not at all) to 7 (definitively/almost always), with higher scores reflecting higher levels of fatigue. Cronbach’s α for the present sample was 0.91.

### Paresthesia

Patients rated their degree of paresthesia on a 10-point visual analogue scale ranging from 0 (no sensations at all) to 10 (severe sensations) [see also Ref. (67)].

### Physical Activity

Physical activity was assessed with the short form of the International Physical Activity Questionnaire (IPAQ-SF). The IPAQ-SF was developed by a working group initiated by the World Health Organization and the Centers for Disease Control and Prevention (83). Based on the results from 12 countries, reliability and validity of IPAQ are comparable to other self-reported measures of physical activity. The IPAQ-SF asks participants about time spent in physical activity over the last 7 days. Minutes of moderate- and vigorous-intensity activities were calculated for the previous week.

### Physical Disability; the Expanded Disability Status Scale (EDSS)

The Expanded Disability Status Scale (EDSS) was employed by a trained neurologist to assess patients’ level of physical disability. The EDSS is an internationally accepted and widely used tool to provide an objective assessment of the disability levels of patients with MS (84, 85). The total score is on a scale from 0 (no impairment in neurological dimensions) to 10 (death due to MS), with increments of 0.5 to 1, and with higher scores reflecting higher levels of disability. Meyer-Moock et al. (86) reported in their systematic review the high validity and reliability of the EDSS. They further concluded that the EDSS is suitable to describe clinical status and physical disability and to monitor disease progression.

### Cognitive Performance

Two instruments were used to measure cognitive performance, the Montreal Cognitive Assessment (MoCA) (87) and the Symbol Digit Modality Test (SDMT) (88). To avoid the learning effect, parallel versions of each tool were used.

#### Global Measurement of Cognitive Performance

The MoCA is a screening instrument that allows a global cognitive measurement to be made through the assessment of a wide range of cognitive functions, such as i) short-term memory; ii) executive functions; iii) visuospatial abilities; iv) language; v) attention, concentration, and working memory; and vi) temporal and spatial orientation. Its extensive validation, international recognition, and recommendation in various guidelines make the MoCA a useful brief cognitive screening tool in both clinical and research contexts (89) (see also https://www.mocatest.org). In the present study, we report the overall score, with higher scores reflecting better cognitive performance.

#### Attention, Concentration, and Information Processing Speed

The Symbol Digit Modality Test (SDMT) (88) is an instrument to assess attention, concentration, and information processing speed. The test consists of single digits paired with nine abstract symbols. Rows of the symbols are arranged pseudo-randomly. The patient must state the number that corresponds to each symbol. The SDMT can be completed within 5 min, including instructions, practice, and testing. Evidence indicating the satisfactory psychometric properties of the SDMT has been provided in previous research (90).

### Exercise Intervention

Regular endurance exercise was undertaken in the course of a standardized 3-week inpatient rehabilitation program. Training consisted of physiologically defined heart-rate-controlled cycling at 60 rpm at the lactate threshold (75% of HR_max_ or 65% of VO2-peak). A training session lasted 30 min, with warm-up and cool-down for the first and last 2 min, respectively. A total of five training sessions were conducted per week, representing standard care at the rehabilitation center. A sport scientist expert in exercise interventions for patients with MS supervised the intervention sessions.

Regular exercise was performed in addition to the normal rehabilitation program that consisted of two physiotherapy interventions per day. Physiotherapy consisted of progressive resistance training (45 min) and a low-intensity physiotherapeutic session (30 min).

### Statistical Analysis

Data were analyzed per protocol. Main outcome variables were changes in objective and subjective sleep, depression, fatigue, and paresthesia. Secondary outcome variables were physical activity and cognitive function.

To compare performances at baseline with performances at study end (3 weeks later), a series of paired *t*-tests was performed. Pearson’s correlations were computed between level of impairment (EDSS score) and sleep, psychological functioning, physical activity, and cognitive performance. The level of significance was set at α ≤ .05. All statistical analyses were performed with SPSS^®^ 25.0 (IBM Corporation, Armonk, NY, USA) for Apple^®^ Mac^®^.

## Results

### Sample Characteristics

Of the 51 patients enrolled at baseline, 46 completed the study (pre/post assessment, and intervention); 5 did not want to participate in the second assessment or left the clinic earlier than scheduled. The mean age of the sample at baseline was 50.74 years (SD = 11.28). In total, 78.4% of the participants were females. There was no difference in age between females (M = 51.89; SD = 11.70) and males [M = 46.00; SD = 8.20; *t*(44) = 1.42, *p* = .16], nor did they differ in EDSS scores [females: M = 5.49, SD = 1.11; males: M = 4.89, SD = 1.27; *t*(44) = 1.41, *p* = .17]. Next, not all participants completed all questionnaires; degrees of freedom therefore vary for the different outcomes. EEG values were available only for 26 patients (14 patients were not willing to participate in the sleep-EEG assessment, and for 6 patients, data were not recorded due to technical failures). EEG completers did not statistically significantly differ from noncompleters as regards age, EDSS, fatigue, paresthesia, cognitive dysfunction (all *t* values < 1.00; *p* values > .50), physical activity, and gender (χ^2^ < 0.5; *p* > .50).

### Objective Sleep

**Table 1** reports the descriptive and inferential statistical indices of objective sleep parameters.

**Table 1:** Objective sleep dimensions at baseline and at the study end.

	Time points		Statistics
	Baseline	Prior to discharge	Paired t test
	M (SD)	M (SD)	
Total sleep time (h:min:s)	6:09:07 (1:21:21)	6:31:44 (1:03:45)	*t*(25) = −1.34, *p* = .19
Sleep efficiency (%)	75.39 (14.12)	82.66 (11.19)	*t*(25) = −2.15, *p* = .04
Sleep onset latency (min)	30.82 (15.63)	7.81 (8.09)	*t*(25) = 6.88, *p* < .01
Sleep period time (h:min:s)	7:27:12 (1:17:22)	7:28:12 (0:58:00)	*t*(25) = −0.07, *p* = .95
Awakenings after sleep onset (*n*)	18.58 (12.6)	15.58 (11.86)	*t*(25) = 1.45, *p* = .16
Wake time after sleep onset (h/min)	1:57:26 (1:08:59)	1:22:30 (1:08:59)	*t*(25) = −2.10, *p* = .04
REM sleep (h:min:s)	1:09:04 (0:41:38)	1:18:02 (0:39:17)	*t*(25) = −1.26, *p* = .22
REM sleep (%)	17.95 (9.82)	19.33 (8.91)	*t*(25) = −0.84, *p* = 41
S1 (h:min:s)	0:32:51 (0:37:01)	0:37:18 (0:46:05)	*t*(25) = −0.53, *p* = .65
S1 (%)	8.88 (9.63)	9.78 (12.41)	*t*(25) = −0.42, *p* = .68
S2 (h:min:s)	3:31:22 (1:25:32)	3:24:51 (1:07:21)	*t*(25) = 0.53, *p* = .65
S2 (%)	54.8 (17.9)	55.12 (18.66)	*t*(25) = −0.15, *p* = .921
S3 (h:min:s)	0:44:45 (0:58:39)	0:45:13 (1:10:18)	*t*(25) = −0.05, *p* = .96
S3 (%)	13.21 (15.80)	10.38 (14.79)	*t*(25) = 1.33, *p* = .19
S4 (h:min:s)	0:16:39 (0:21:43)	0:20:30 (0:24:04)	*t*(25) = −0.96, *p* = .35
S4 (%)	4.1 (5.16)	4.76 (5.47)	*t*(綤25) = −0.73, *p* = .47
Light sleep (h:min:s)	3:59:21 (1:02:13)	3:55:27 (1:07:14)	*t*(25) = 0.30, *p* = .77
Light sleep (%)	63.59 (14.48)	63.58 (17.96)	*t*(25) = 0.01, *p* = .99
Deep sleep (h:min:s)	1:08:43 (1:20:54)	1:13:03 (1:34:49)	*t*(25) = −0.38, *p* = .71
Deep sleep (%)	17.45 (18.90)	16.27 (18.87)	*t*(25) = 0.55, *p* = .58

S, stage; REM, rapid eye movement; light sleep, Stage 1 + Stage 2; deep sleep, Stage 3 + Stage 4.

With regard to sleep continuity, sleep onset latency significantly decreased, sleep efficacy improved, and the time of awakenings after sleep onset diminished. For all other dimensions of sleep continuity (total sleep time, sleep period time, and numbers of awakenings after sleep onset), there were no significant differences between the baseline and the study end. With regard to sleep architecture (stages 1–4 and REM-sleep: min and %), there were no significant differences.

### Subjective Sleep, Depression, Fatigue, and Paresthesia

**Table 2** reports the descriptive and inferential statistical indices for subjective sleep, depression, fatigue, and paresthesia. These statistical indices are not therefore repeated in the text. From baseline to the end of the study, subjective sleep (complaints), depression, fatigue, and paresthesia decreased significantly.

**Table 2:** Descriptive and inferential statistical overview of values for subjective sleep, depression, fatigue, and paresthesia at baseline and at the study end.

	Time points		Statistics
	Baseline	Study end	Paired t test
	M (SD)	M (SD)	
Sleep complaints (ISI)	15.18 (5.71)	14.00 (4.59)	*t*(44) = 2.03, *p* = .048
Depression (BDI fast screen)	2.87 (2.62)	1.85 (2.04)	*t*(45) = 3.48, *p* = .001
Fatigue (FSS)	42.18 (14.34)	36.11 (15.92)	*t*(43) = 3.29, *p* = .002
Paresthesia	4.15 (2.90)	3.26 (2.51)	*t*(45) = 2.75, *p* = .009

ISI, Insomnia Severity Index; BDI, Beck Depression Inventory; FSS, Fatigue Severity Scale.

### Subjective Physical Activity

**Table 3** reports the descriptive and inferential statistical indices for subjective physical activity. From baseline to the study end, subjective vigorous and moderate physical activity increased significantly.

**Table 3:** Descriptive and inferential statistical overview of values for subjective physical activity and cognitive performance at baseline and at the study end.

	Time points		Statistics
	Baseline	Study end	Paired t tests
	M (SD)	M (SD)	
IPAQ moderate	155.00 (229.78)	689.4 (942.58)	*t*(42) = 3.26, *p* = .01
IPAQ vigorous	98.64 (223.98)	559.09 (1,161.41)	*t*(42) = 2.32, *p* = .03
SDMT	33.81 (10.58)	36.51 (11.56)	*t*(42) = −2.31, *p* = .03
MoCA sum	26.16 (2.45)	27.15 (2.83)	*t*(ჼ40) = −2.60, *p* = .01

IPAQ, International Physical Activity Questionnaire; MoCA, Montreal Cognitive Assessment; SDMT, Symbol Digit Modality Test.

### Cognitive Performance

**Table 3** also provides the descriptive and inferential statistical indices for cognitive performance. From baseline to the study end, attention, concentration, and information processing speed (SDMT), as well as global cognitive performance (MoCA), significantly improved.

### Correlations Between Degree of Impairment (Expanded Disability Status Scale Scores) and Objective and Subjective Sleep, Depression, Fatigue, Paresthesia, Subjective Physical Activity, and Cognitive Performances

All correlation coefficients were small (all *r* values < .15) and nonsignificant (all *p* values > .30). In other words, degree of physical impairment (EDSS) was not related to objective or subjective sleep, depression, fatigue, paresthesia, subjective physical activity, or cognitive performances either at baseline or at study end.

## Discussion

The key findings of the present study were that in a sample of patients with MS, a 3-week exercising program led to improvements in both objective and subjective sleep, depression, fatigue, paresthesia, and cognitive performance. The pattern of the result makes an important contribution to the current literature as, to the best of our knowledge, this is the first study to show that regular exercising has positive effects on both the objective and subjective sleep of MS patients.

Four hypotheses were formulated and these are considered in turn.

Following others (51, 52, 54), our first hypotheses was that a 3-week program of regular exercise would benefit objective sleep, and this hypothesis was confirmed. Specifically, sleep efficiency improved, sleep onset latency shortened, and the wake time after sleep onset decreased; thus, improvements in sleep continuity were observed, though we found no significant changes in sleep architecture. We believe that these findings add to the current literature in an important way as, to the best of our knowledge, these effects have not previously been investigated or observed among patients with MS.

We also note that, at baseline, indices of sleep quality such as sleep efficiency (75.4%) and sleep onset latency (about 31 min) had particularly low values. In our opinion, these findings are consistent with observations that sleep quality deteriorates with the progression of MS (3, 4, 6, 7, 29, 31, 42), while in studies by Sadeghi Bahmani and colleagues (20, 21), sleep complaints were not found at disease onset or 2 years later.

Our second hypothesis, following Siengsukon et al. (56), was that a 3-week program of regular exercise would improve subjective sleep, and this was confirmed. These findings therefore replicate those from the only previous study of which we are aware, that by Siengsukon et al. (56). However, we expanded upon their study, in finding significant changes in subjective sleep within a 3-week period.

Several studies have shown that physical activity has the potential to ameliorate sleep complaints both in healthy individuals and in patients with chronic diseases. Of various possible explanations, the most plausible are the consequences of reduced physical tiredness and rumination and enhanced mood in producing improvement in sleep complaints following a program of physical activity (51, 53).

Our third hypothesis was that a 3-week program of regular exercise would have a positive impact on the symptoms of depression, fatigue, and paresthesia of MS patients. This was confirmed for all three variables. Similar effects for depression have been reported in other studies. For example, Mota-Pereira et al. (91) showed that a program of physical activity involving regular walking for 12 consecutive weeks reduced symptoms of depression in a sample of treatment-resistant patients with major depressive disorders. In this respect, several meta-analyses have concluded that regular physical activity has potential benefits with respect to symptoms of depression both among patients with mental impairments (92–98) and among patients with MS (67). As regards fatigue, our results are consistent with findings from previous studies of MS patients (24, 57, 99–101), though we add to the current literature in demonstrating such improvements within a period of 3 weeks of regular exercising.

As regards paresthesia, so far only one study, Razazian et al. (67), has focused on improvements with respect to this characteristic following an intervention involving physical activity intervention. They showed that both a 2-month yoga and a 2-month aquatic exercise intervention reduced paresthesia in MS patients. A closer inspection of their findings reveals these improvements were due to reductions in depression.

Our fourth hypothesis was that a 3-week program of regular exercise would lead to improvements in cognitive function, and this was supported. These results therefore match the conclusions of a review by Sandroff et al. (102) analyzing the impact of physical activity on cognitive performance in MS patients across 26 studies. However, these authors also found that not all subtests significantly changed over time. Similarly, for results based on the MoCA assessment, we found significant effects with respect to memory and language, but not executive function, visuospatial ability, attention, or abstract thinking. That can be explained in terms of the type of cognitive skill, the timing of the intervention, the type of intervention, and the severity of the disease. Results equivalent to those for MoCA were obtained for SDMT. We were able to show first that, as in other studies, SDMT is a very precise tool for assessing cognitive performance in patients with MS. Second, changes in cognitive function were more related to the total score for cognitive performance than to specific skills. After all, all cognitive abilities are influenced by other skills, and what we need in coping with daily life is more related to the overall cognitive ability than to individual cognitive skills. Our findings indicate that a 3-week program of regular exercise is useful in enhancing general cognition, although the short period of this intervention may have been responsible for the lack of improvement in every aspect of cognition.

Despite the novelty of the present findings, several limitations warrant against their overgeneralization. The main limitation of this study is the absence of a control group. Given that the exercise program was part of a defined schedule for all patients hospitalized at the Valens Rehabilitation Center, including a control group without an exercise program was not an option. Nevertheless, we believe that the present pattern of results is robust, given that changes could be observed across a broad range of characteristics (objective and subjective sleep, psychological functioning, cognition). Second, it is possible that the pattern of results might have arisen because further unassessed variables biased two or more dimensions in the same or the opposite directions. Third, bias is also possible in the sample as, for reasons related to health insurance conditions, not all patients with MS are admitted to the rehabilitation center. Fourth, as regards depression, we relied on self-reports, while experts’ ratings would have further strengthened the present the pattern of the results. Fifth, a follow-up study could provide more insight into the longer-term effects of such interventions once patients have returned home and to their normal working and social environments.

## Conclusions

A 3-week exercise program for patients with MS improved both objective and subjective sleep, along with improvements in psychological functioning and cognitive performance.

## Ethics Statement

The local ethics committees of Basel (Ethikkommission Nordwestschweiz; EKNZ: 2016-01347) and St. Gallen (EKOS; Switzerland) approved the study.

## Author Contributions

Study design: DSB, JK, JB, MP, UP, VS, MG, EHT, and SB. Data gathering: DSB, JK, JB, and MP. Statistics: DSB, MG, VS, EHT, and SB. Manuscript draft: DSB, JK, JB, MG, EHT, and SB. Approved final version: DSB, JK, JB, MP, UP, VS, MG, EHT, and SB.

## Conflict of Interest Statement

The authors declare that the research was conducted in the absence of any commercial or financial relationships that could be construed as a potential conflict of interest.
